# Supplementary material for: Correlation analysis of serum TLR4 protein levels and TLR4 gene polymorphisms in gouty arthritis patients
Source: PLoS One. 2024 Apr 23;19(4):e0300582. doi: 10.1371/journal.pone.0300582 (PMC11037531; doi:10.1371/journal.pone.0300582)
Supplement: Attachment — Submitted filename: Response to Reviewers.docx [file pone.0300582.s001.docx]

Thank you for your letter and for reviewer’ comments concerning our manuscript entitled “Correlation analysis of serum TLR4 protein levels and TLR4 gene polymorphisms in gouty arthritis patients”. These comments are all valuable and very helpful for revising and improving our paper, as well as the important guiding significance to our researches. We have studied comments carefully and have made corrections which we hope meet with approval. Our response is given in normal font and changes to the manuscript are given in the rad text. The responds to the comments are as follows:

1. With regard to "provide additional details regarding participant consent", informed consent of the participants in this study has been added to the methodology in the text as follows: The participants included in this study were all adult males, and there were no minors, who were informed of the informed consent at the time of inclusion. The participants included in this study were all adult males, with no minors, and were informed of the informed consent form at the time of inclusion, and were included in the study after verbal consent was given by the participants.
2. Role of Funder statement: Natural Science Foundation of Xinjiang(2022D01C756) recipient is Lu Liu, Natural Science Foundation of Xinjiang(2020D01C263) and Natural Science Foundation of China (82002276) recipient is Xiaobin Guo, Natural Science Foundation of China (81460153) recipient is Lei Miao. All three of the above grantees participated in this study, and their contributions are described in the Author Contributions section, and they are all credited in this manuscript.
3. According to the statement of financial support for manuscripts that have been deleted at your request.
4. The data availability statement you mentioned, all the authors of this study agree to data sharing, we will share the data according to your request, but we would like to set up access restrictions to our research data before acceptance of the paper.

**Response to Reviewer #1's comments:**

1. Regarding your suggestion that some references to the results of previous generations are missing in the introduction, we have added references and labeled them in the revised draft.
2. The suggestion that the legends in the figures and tables should indicate the number of study participants sampled in each group has been modified and is indicated in the revised draft.
3. Regarding "The statistical tests used in each figure and table must be described in the legend of the figure/table", we have revised and labeled the figures and tables in the manuscript if they need to be revised.
4. Regarding your suggestion that in the discussion section it is not clear what the authors mean by “non-specific immune cells”, this denomination has been removed from the manuscript.

**Response to Reviewer #2's comments:**

1. The question "What are the specific requirements for the quality and concentration of DNA in the samples for this study?" has been modified and added to the manuscript in section 2.4 Whole blood DNA extraction testing. We have modified this statement and added it to the "2.4 Whole blood DNA extraction testing" section of the manuscript.
2. Regarding "What was the basis for the selection of the rs2149356, rs2737191 and rs10759932 loci of the TLR4 gene in this study?" We have supplemented the manuscript with 2.5 SNP selection in the TLR4 gene and primer synthesis section.
3. In response to the question "Is it appropriate to have a separate list of information in Table 1?", we have deleted Table 1 and labeled it in the revised version.
4. Regarding "Please add the manufacturer and product numbers of the primary kits used in this study." We have added this to the "2.2 Reagents and disposables" section of the manuscript.
5. Why were the genotyping results not verified by sampling and sequencing? The genotyping in this study was not validated by sequencing, but the genotyping of the three loci studied in this paper was performed using the Multiple Point Mutation Rapid Test, which has the advantage of being easy to perform and highly sensitive.
6. Regarding "Why did the authors not choose two-tailed tests in their statistical analysis?", the two-tailed test was used for all analyses involving variability in this study, and has been added to the "2.8 Statistical analysis" section.
7. We have combined Tables 3, 4, and 5 into one table in the Table 2 section of the revised draft and have highlighted them in red.
8. Regarding "Do the three loci of the TLR4 gene in this study fulfill Hardy-Weinberg equilibrium?" The present study fulfills the Hardy-Weinberg equilibrium test and is supplemented in "2.5 SNP selection in the TLR4 gene and primer synthesis" in the manuscript.
9. Regarding the question "Is it appropriate that the object of study in this paper is limited to the population of one region and the conclusion does not emphasize a specific population?" The object of this study are coming to a region, there may be poor population extrapolation, but the authors of this paper this study still has its significance, the region's population distribution is not limited to one ethnic group, is a multi-region, multi-ethnic inclusion of the provinces and cities, although the investigation of the data is a region, but there is no China's population distribution there are large differences.
